# Prevalence and Correlates of Mental Health Problems among Chinese Adolescents with Frequent Peer Victimization Experiences

**DOI:** 10.3390/children8050403

**Published:** 2021-05-17

**Authors:** Liuyue Huang, Kaixin Liang, Weiwei Jiang, Qiaomin Huang, Na Gong, Xinli Chi

**Affiliations:** 1 School of Psychology, Shenzhen University, Shenzhen 518061, China; 1910481006@email.szu.edu.cn (L.H.); liangkaixin2020@email.szu.edu.cn (K.L.); 2070481022@email.szu.edu.cn (W.J.); 2070481032@email.szu.edu.cn (N.G.); 2 Law School of Shenzhen University, Shenzhen 518060, China; 1810151024@email.szu.edu.cn

**Keywords:** adolescents, anxiety, depression, insomnia, peer victimization

## Abstract

This study investigated the prevalence and correlates of mental health outcomes, particularly depression, anxiety, and insomnia, in adolescents with frequent peer victimization experiences (FPVEs). In this cross-sectional study, 490 adolescents reported having FPVEs (prevalence, 4.2%; mean age, 13.40 ± 1.38 years old; 52.2% male) completed a series of surveys to evaluate their demographic factors and mental health status. The results showed that the prevalence of depression, anxiety, and insomnia were 50.3%, 33.8%, and 40.2%, respectively. Older age, being female, being left behind, and more adverse childhood experiences were correlated with more symptoms of depression, anxiety, and insomnia among adolescents with FPVEs. At the same time, social support and self-compassion were good for ameliorating these mental health problems. Implications for intervention programs designed to improve the mental health of adolescents with FPVEs were also discussed.

## 1. Introduction

Peer victimization refers to physical, verbal, or interpersonal attacks (e.g., physical violence, threats, teasing, insults) on individuals by peers [1]. Peer victimization has many negative influences on adolescents, including psychological/social maladaptation, loneliness, anxiety, depression [2,3,4], chronic health conditions [5], and even suicide [6]. Nevertheless, peer victimization is widespread among adolescents. Around 150 million youth aged 13–15 have experienced peer violence worldwide [7]. Related studies show that approximately 7.7–9% of adolescents suffer from frequent peer victimization experiences (FPVEs) worldwide [8,9]. A recent study conducted in Finland that evaluated 4447 adolescents found that 20% of the participants reported being frequently victimized by peers [10]. Similar studies conducted in China have found that approximately 2–17% of adolescents reported FPVEs [11,12]. However, these studies were more than ten years ago, and much has changed in China’s socio-economic conditions since then. Compared to other countries, the prevalence of FPVEs and associated mental health problems (e.g., depression and anxiety) in adolescents with FPVEs are still less known in China. Thus, this study aimed to examine the prevalence of FPVEs among Chinese adolescents and the prevalence of associated mental health outcomes (i.e., depression, anxiety, and insomnia) among this cohort.

Considering the psychological impacts of FPVEs, it is critical to clarify both the risk factors and protective factors associated with mental health problems. The dynamic stress-vulnerability model [13] suggests that many factors can be potential risk factors for developing disorders such as depression, anxiety, and insomnia. These factors may be demographic (e.g., age and gender), social (e.g., parental education, family structure, family income, left-behind status, and social support), or psychological (e.g., adverse childhood experiences [ACEs], positive youth development [PYD], and self-compassion [SC]). Thus, this study will explore these factors related to mental health problems using the dynamic stress-vulnerability model.

Previous studies have shown there were gender-based differences in the psychological problems adolescents with FPVEs developed. Female adolescents with FPVEs reported more psychological problems compared to their male peers [14,15]. Age was also an essential potential influencing factor. Compared to young children, adolescents attach more importance to peer relationships [16], so peer victimization during this period may cause a greater variety of and more severe adverse effects on their mental health [11]. However, whether such differences regarding gender and age remain the same among adolescents with FPVEs is still being investigated.

Social factors also play an essential role in the development of psychological problems among adolescents with FPVEs. Existed studies have revealed several factors that may put adolescents at increased risk of developing mental health problems, including mothers with less education [17], having divorced parents [18], having siblings [19], being from a low-income family [20], being left behind by parents who are migrant workers for more than six months [21], and inadequate social support [22]. Left-behind children are of particular concern because they may lack parental care and support, which can adversely influence their psychosocial adaptation and mental health [23]. Many studies have also found that adolescents with a history of FPVEs were less likely to make friends in college and more likely to be socially isolated; such a lack of social support is also a contributing factor to mental health problems [24].

As for psychological factors, ACEs could lead to developmental maladjustment during adolescence and increased psychological vulnerability to mental illness [13,25]. A growing body of evidence indicates that risk factors (e.g., childhood trauma) for mental health problems can be cumulative [26]. Adolescents who report experiencing FPVEs may also have experienced other ACEs (e.g., emotional abuse by parents) simultaneously [27]. Cumulative risk factors may have severe negative impacts on the mental health of adolescents with FPVEs, according to developmental psychopathology [28]. Furthermore, PYD qualities could positively predict psychological wellbeing and negatively predict mental problems in adolescents [29,30], although Chinese adolescents with FPVEs experience the same effect are yet to be examined. Notably, SC could significantly mitigate the influence of adverse events and promote psychological wellbeing [31,32]. Individuals with a high level of SC may treat themselves with kindness in suffering [33], whereas those with a low level of SC may develop more severe mental health problems [34].

As mentioned above, the mental health of adolescents with FPVEs is affected by many factors. Existing research has reported some critical risk and protective factors for individuals with FPVEs, which provides theoretical support for developing practical intervention studies. However, there are some research gaps concerning this topic that have yet to be addressed. First, studies of the mental health of adolescents with FPVEs are still limited in China. So far, most of the studies on FPVEs have been carried out in Western culture; less attention has been paid to how this problem affects Chinese youth [2]. Second, most studies focus specifically on demographic factors [35,36], but social and psychological factors are also important factors influencing the mental health of adolescents with FPVEs. This study aims to fill these gaps.

The first aim of the present study is to determine the prevalence of FPVEs among Chinese adolescents. The second aim is to investigate the prevalence of mental health problems (specifically depression, anxiety, and insomnia symptoms) in Chinese adolescents with FPVEs. The third aim is to use the dynamic stress-vulnerability model to investigate demographic (i.e., age and gender), social (i.e., parental education, family structure, number of children in the family, family income, left-behind status, and social support), and psychological (i.e., ACEs, PYD, and SC) factors associated with mental health problems among adolescents with FPVEs. The results of this study may offer detailed insights into the psychological health status and influencing factors of adolescents with FPVEs, and will thus constitute an empirical basis for developing new prevention and intervention practices.

## 2. Methods

### 2.1. Study Participants and Procedure

Data for the present study were part of the Guangxi Youth Mental Health Survey (GYMHS). GYMHS is a project developed by the Guangxi Education Bureau in collaboration with Shenzhen University, conducted in May 2020 in China. It was designed to help Guangxi Region assess the mental health status and protective/risk factors among local students during the COVID-19 pandemic. GYMHS was a relatively low-cost and school-based survey using online and self-administered questionnaires to obtain data. In the survey, 31 schools (15 primary schools, 13 junior middle schools, and three senior high schools) were randomly selected in which all students were invited. With the assistance of psychological consultants in local schools, graduate students of psychology carried out the study. Before collecting data, the primary purpose of this research was illustrated to all participants, followed by gaining their informed consents and parental assent forms.

**Figure 1:**
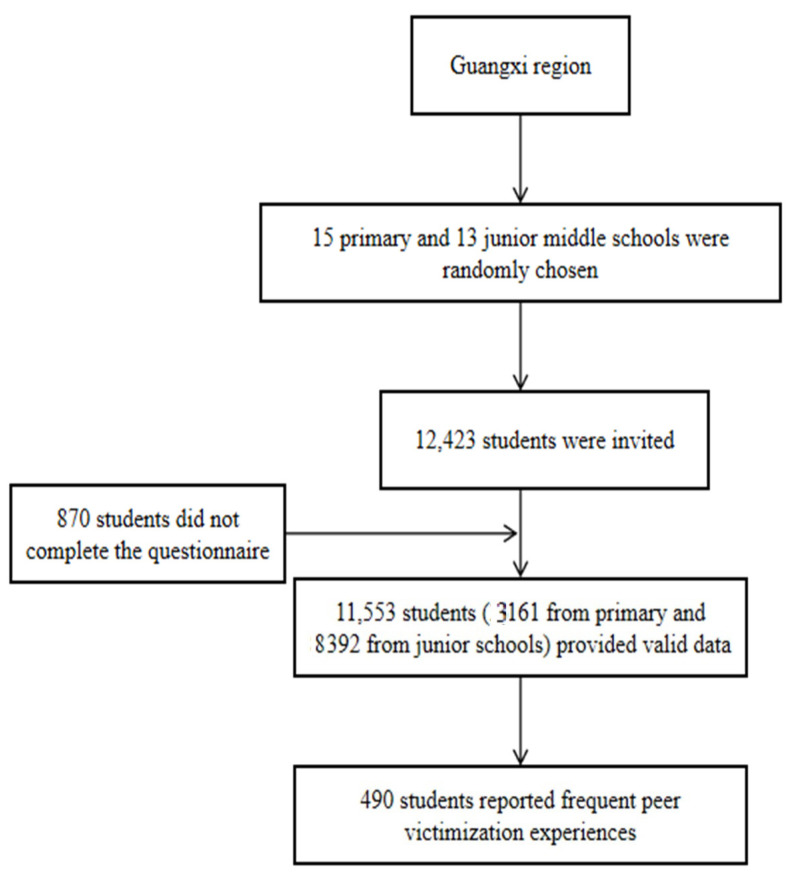
Flow chart of sampling and participant recruitment.

Data of the current study came from the survey of primary and junior high schools in GYMHS. A total of 11,553 students (3161 from primary school [senior pupils over nine years old] and 8392 from junior school) completed the survey and consisted of the analytical sample, at a response rate of 93.9%. Four hundred and ninety students (comprising 4.2% of all respondents) replied positively to the item in the *Adverse Childhood Experience Scale*-*Revision* that ascertained whether a respondent had been subject to an FPVE before 18 years old. In other words, the item asked whether the respondent had been frequently or very frequently abused by other children (including siblings) verbally or physically [37]. These respondents were selected for further statistical analysis in this study. The procedure of sampling and participant recruitment is presented in Figure 1. To maximize the effectiveness of self-report, the anonymity and confidentiality of all participants were guaranteed. The Shenzhen University Ethics Committee approved this research project (no. 2020005).

### 2.2. Measurement

#### 2.2.1. Dependent Variables

##### Depression Symptoms

The Chinese version of the 9-item Patient Health Questionnaire (PHQ-9) was used to assess experiences of depression in the preceding two weeks. This instrument consisted of nine items, each rated on a four-point Likert scale ranging from 0 (not at all) to 3 (nearly every day). The instrument was validated and administered to Chinese youth in previous research [38]. The highest score for the nine items combined was 27, with higher scores indicating more severe depression symptoms. The severity of depression symptoms can be classified based on PHQ-9 scores: 0–4 (minimal), 5–9 (depression), 10–14 (moderate), 15–19 (moderately severe), and 20–27 (severe). According to previous studies, individuals with a total score of 10 or above were considered as having probable major depression [39]. The Cronbach’s α of the PHQ-9 was 0.92.

##### Anxiety Symptoms

Anxiety symptoms were measured using the 7-item Generalized Anxiety Disorder Scale (GAD-7) [40]. The GAD-7 was validated and used in the Chinese population in previous studies [41,42]. This instrument consists of seven items, with each item rated on a four-point Likert scale (0 = Not at all to 3 = Nearly every day). A higher mean score reflected more severe anxiety symptoms. The severity of anxiety can be classified as minimal (0–4), mild (5–9), moderate (10–14), and severe (15–21). Based on previous studies, a cut-off score of 10 was adopted as indicating the presence of moderate to severe anxiety symptoms in this study [43]. The Cronbach’s α of the GAD-7 was 0.93.

##### Insomnia Symptoms

Insomnia symptoms were measured using the Chinese version of the 8-item Youth Self-rated Insomnia Scale (YSIS), with each item rated on a five-point Likert scale. The sum of the scores for all eight items yielded a total YSIS score ranging from eight to 40. A higher total score indicated greater severity of insomnia symptoms during the preceding month. Participants who scored less than 22 were classified as having minimal insomnia symptoms, 22–25 mild insomnia, 26–29 moderate insomnia, and ≥ 30 severe insomnia [44]. For individual scored ≥ 26 was classified as clinical insomnia. The Cronbach’s α of the YSIS was 0.91.

#### 2.2.2. Independent Variables

##### Sociodemographic Correlates

Participants were requested to report on their age, gender (0 = male, 1 = female), family structure (0 = intact, 1 = other), residence (0 = urban, 1 = rural), migrant status (0 = non-migrant, 1 = migrant), siblings (1 = yes, 2 = no), parents’ education (1 = junior school and below, 2 = senior school, 3 = bachelor’s degree, 4 = above bachelor’s degree), left-behind status (0 = no, 1 = yes), and monthly household income per capita in renminbi (RMB) (1 = “below 1000”, 2 = “1000–1999”, 3 = “2000–2999”, 4 = “3000–3999”, 5 = “4000–4999”, 6 = “5000–5999”, 7 = “above 6000”).

##### Social Support

We used the Chinese version of the social support rating scale (SSRS) to assess levels of social support. SSRS includes three dimensions: subjective support, objective support, and utilization of support, with a total of ten items. The scores of all ten items were combined to obtain the total score. A higher total score represented a higher level of social support. The SSRS has good reliability and validity and has been validated and used in a Chinese context [45,46]. In the current study, Cronbach’s α of the scale was 0.84.

##### Adverse Childhood Experiences (ACEs)

ACEs were assessed using the Chinese version of the revised Adverse Childhood Experiences Scale [37]. Each ACE was coded based on the presence (coded as “1”) or absence (coded as “0”) of each experience in the student’s childhood. The prevalence and types of adverse childhood experiences are shown in Table 1. An overall ACE score, ranging from 0 to 14, was created by summing the number of ACEs assessment items that received a positive response. The Chinese version of the revised Adverse Childhood Experiences Scale has good reliability and validity [47]. The Cronbach’s α of the scale was 0.77 in the current study.

##### Self-Compassion

The Chinese version of the Self-compassion Scale-Short Form (SCS-SF) was used to measure SC [48]. It consisted of 12 items, with each item rated on a five-point Likert scale (1= rarely to 5 = almost always). After the reversed-coding questions were revised, the total scores ranged from 12 to 60, with higher scores indicating stronger SC. The Cronbach’s α of the scale was 0.86 in the current study.

##### Positive Youth Development

The concise measure of the Five Cs of Positive Youth Development (PYD-VSF) was used to measure psychosocial competence [49]. The adapted Chinese version contained 17 items that assessed five dimensions (competence, confidence, character, caring, and connection). Examples of these items included “I am happy with myself most of the time” (confidence) and “When I see someone being taken advantage of, I want to help them” (caring/compassion). Each item was rated on a five-point Likert scale (1 = strongly agree to 5 = strongly disagree). The score on the negative item (item 6) was reversed when added to the other items, with higher scores indicating more positive development. The Cronbach’s α of the scale was 0.86 in the current study.

### 2.3. Statistical Analyses

**Figure 2:**
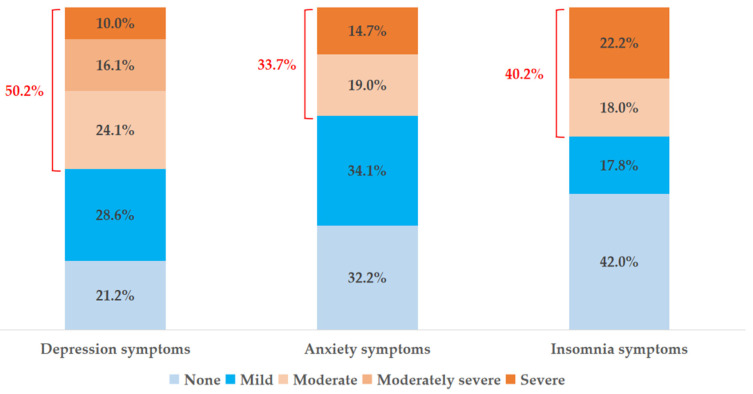
The prevalence of different severities of depression, anxiety, and insomnia symptoms in Chinese adolescents with frequent peer victimization experiences.

First, we selected the subjects who screened positive on the item concerning FPVEs for further statistical analysis and calculated the prevalence rate of FPVEs. Frequencies with percentages or means with standard deviations (SDs) of demographic variables were also computed (i.e., gender, age, family size, residence, migrant status, family structure, parents’ education, left-behind status, family income, and ACEs) (see Table 1). The prevalence of different severities of depression, anxiety, and insomnia symptoms in adolescents with FPVEs were calculated (see Figure 2). Next, to explore how demographic, social, and psychological factors were associated with mental health outcomes (i.e., depression, anxiety, and insomnia), we performed three-step hierarchical linear regression analyses. The first step dealt with demographic factors (i.e., age and gender). In the second step, social factors (i.e., father’s education, mother’s education, family structure, number of siblings, family income, left-behind status, and social support) were considered independent variables. Last, psychological factors (i.e., ACEs, PYD, and SC) were addressed in the third step (see Table 2). All analyses were performed using the Statistical Package for the Social Sciences (SPSS) for Windows, version 23.0. The statistical significance was set at *p* < 0.05 (two-tailed) in the interpretation of the results.

## 3. Results

### 3.1. Characteristics of Participants

The final sample comprised 490 subjects (mean age = 13.40  ±  1.38; age range: 11–16 years old). Of these, 47.8% were girls, and 52.2% were boys. The prevalence rate of FPVEs was 4.2%. The majority of participants reported having one or more siblings (81.4%) and being from a rural area (78.6%). Around half of the subjects reported being left-behind children (42.4%). Most participants reported their parents’ education level as being junior high school or below. For adolescents with PFVEs, 63.1% and 50.0% concurrently experienced frequent or persistent emotional abuse and emotional neglect by parents or other adults, respectively. The prevalence of physical abuse by parent(s) or other adults was also relatively high (32.0%) among the adolescents with PFVEs. The percentage of adolescents with FPVEs who reported concurrent ACEs and the numbers of concurrent ACEs were startling: 35.1% reported having five or more ACEs. More information reported by the study participants is presented in Table 1.

### 3.2. Prevalence of Depression, Anxiety and Insomnia Symptoms in Adolescents with FPVEs

The Mean and SD of depression, anxiety, and insomnia symptoms among adolescents with FPVEs were 10.51 ± 6.70, 7.74 ± 5.17, and 23.08 ± 8.13, respectively. Regarding the prevalence of depression, 21.2% reported minimal depression symptoms, while 28.6%, 24.1%, 16.1%, and 10.0% of participants reported mild, moderate, moderately severe, and severe depression symptoms, respectively. As for anxiety symptoms, 32.2% of participants showed minimal anxiety symptoms, while 34.1, 19.0%, and 14.7% of participants reported mild, moderate, and severe anxiety symptoms, respectively. Regarding insomnia, 42.0% of participants showed minimal insomnia symptoms, whereas 17.8%, 18.0%, and 22.2% had mild, moderate, and severe insomnia symptoms. In summary, 50.2% (PHQ-9 ≥ 10), 33.7% (GAD-7 ≥ 10), and 42.0% (YSIS ≥ 26) of adolescents were screened positively on depression, anxiety, and insomnia, respectively, based on the recommended cutoff point. Further information is presented in Figure 2.

### 3.3. Predictors of Mental Health Outcomes

#### 3.3.1. Depression

With regard to demographic factors, older age (*β* = 0.19, *p* < 0.001) and female gender (*β* = 0.20, *p* < 0.001) were significantly associated with a higher likelihood of depression symptoms. Regarding social factors, being left behind children (*β* = −0.12, *p* < 0.01) and low social support (*β* = −0.33, *p* < 0.001) were found to positively predict depression symptoms among adolescents with FPVEs. After controlling for demographic and social factors, psychological vulnerabilities (i.e., more ACEs and lower PYD and self-compassion) were also significantly associated with depression symptoms. Specifically, more ACEs (*β* = 0.13, *p* < 0.01) and weak SC (*β* = −0.38, *p* < 0.001) were related to more severe depression symptoms. Parents’ education, family structure, siblings, family income, and PYD were not significantly correlated with depression symptoms in our study (Table 2).

#### 3.3.2. Anxiety

The demographic factors of age (*β* = 0.14, *p* < 0.01) and gender (*β* = 0.17, *p* < 0.001) were found to be predictors of anxiety. Regarding social factors, mother education can predict anxiety symptoms (*β* = 0.15, *p* < 0.01). Likewise, being left behind (*β* = −0.12, *p* < 0.01) and low social support (*β* = −0.28, *p* < 0.001) positively predicted anxiety symptoms among adolescents with FPVEs. After controlling for demographic and social factors, psychological factors remained to be significantly associated with anxiety symptoms. Specifically, more ACEs (*β* = 0.16, *p* < 0.001) and weak SC (*β* = −0.37, *p* < 0.001) were related to more severe anxiety symptoms. Family structure, siblings, family income, and PYD were not significantly correlated with anxiety symptoms in our study (Table 2).

#### 3.3.3. Insomnia

For demographic factors, age (*β* = 0.22, *p* < 0.001) and gender (*β* = 0.23, *p* < 0.001) were associated with insomnia symptoms. With regard to social factors, being left behind (*β* = −0.08, *p* < 0.05) and low social support (*β* = −0.35, *p* < 0.001) positively associated with insomnia symptoms among adolescents with FPVEs. After controlling for demographic and social factors, psychological factors were also significantly associated with insomnia symptoms. More ACEs (*β* = 0.09, *p* < 0.05) and weak SC (*β* = −0.26, *p* < 0.001) were associated with more severe insomnia symptoms (see Table 2).

## 4. Discussion and Conclusions

The present study investigated the prevalence of FPVEs and symptoms of depression, anxiety, and insomnia among Chinese adolescents with FPVEs. The risk and protective factors for these mental health outcomes were explored and analyzed, and the interpretation of the corresponding results is below.

The prevalence of FPVEs was 4.2%, a rate lower than that found in previous studies in Western contexts, which showed a prevalence of 5–15% in children and adolescents experiencing severe or persistent peer abuse [10,50]. The difference in these specific rates may be related to the participants’ cultural background and age, and the research methods. Another more direct reason is the classification criteria used, as this study focuses on the frequent but not occasional experiences of FPVEs. As mentioned above, domestic studies conducted more than ten years ago found that approximately 2–17% of adolescents in primary and secondary schools in China reported FPVEs [11,12]. These results indicate that bullying has been a common problem among adolescents across cultures for decades.

Meanwhile, the prevalence of mental health problems was high in this sample population, with 50.3%, 33.8%, and 40.2% of participants reporting depression, anxiety, and insomnia, respectively. Notably, other ACEs were highly prevalent among adolescents with FPVEs: 63.1%, 50.0%, and 32.0% of participants reported being subjected to frequent or persistent emotional abuse, emotional neglect, and physical abuse, respectively. More than half of the participants reported experiencing five or more ACEs other than (and in addition to) FPVEs, indicating that adolescents with FPVEs faced multiple stressors. Such concurrences of ACEs may partially account for the high prevalence of depression, anxiety, and insomnia symptoms in adolescents with FPVEs. These findings indicate that the psychological wellbeing of adolescents with FPVEs is particularly vulnerable: This unique group of children is in a precarious situation and needs special attention.

We also identified some other factors related to the severity of mental health problems. About demographic factors, age and gender could affect all psychological outcomes. Specifically, older adolescents reported more mental health problems, consistent with a previous study [51]. One possible explanation may be that peer relationships are more important to older adolescents, and thus being subjected to peer victimization may lead to more severe problems. Girls with FPVEs were more likely to suffer from more severe depression, anxiety, and insomnia than boys. This result confirms Klomek et al.’s finding that female victims of peer victimization were more prone to develop internalized problems (e.g., depression) [9]. Another possible reason might be that, compared to the peer victimization boys experienced, girls were more likely to be subjected to social exclusion and relational victimization [52,53], which may have more severe psychological consequences [54].

Concerning social factors, there are some common predictors for depression, anxiety, and insomnia. The results indicated that being left behind positively predicted three mental health problems among adolescents with FPVEs, consistent with previous studies conducted among left-behind children in China and around the world [55,56,57]. The reasons may be that left-behind children are less supervised, protected, and cared for by their parents, which may increase their risk of peer victimization [58] and promote psychological problems. The long separation from their parents may also mean adolescents with FPVEs lack sufficient support for recovery [59]. Thirdly, physical inaccessibility and lack of communication with parents may disrupt parent-child attachment, detrimental to emotional regulation [60]. We also found that more social support predicted fewer mental health problems, which confirmed previous findings [61,62]. According to the main effect model of social support, social support may directly protect an individual when experiencing stressful events [63]. Thus, the adverse effects of FPVEs could be buffered by social support among adolescents with FPVEs.

Compared to demographic and social factors, psychological factors proved to be stronger predictors of mental symptoms among adolescents with FPVEs. More ACEs were associated with more severe depression, anxiety, and insomnia among adolescents with FPVEs, consistent with past studies and developmental psychopathology theory, as mentioned above [28]. One explanation may be that these ACEs interrupted the normal development of adolescence, leading to mental health problems [64,65]. We also found that weak SC was a decisive risk factor for mental health problems, which supported the previous studies [66,67]. SC, referring to treating oneself kindly and mindfully in difficulties, is an essential emotional regulation skill when an individual is distressed. Thus, weak SC is often associated with less psychological resilience and may increase vulnerability to emotional disorders.

Several limitations must be noted regarding the present study. The first concerns the participants who took part in the study, most of whom are from Guangxi Province, in southern China: Because Guangxi is an economically underdeveloped province, this prevents generalization of the results. Future studies can carry out similar studies in other areas to provide further evidence to design relevant interventions and policies. Another limitation is that we adopted dichotomous response items (none and frequent/extremely frequent) in the questionnaire, which may leave out participants who experienced some peer victimization but would not describe it as frequent. Third, the study used a cross-sectional design, so longitudinal studies are needed to precisely portray the causality of associated factors and the symptoms of depression, anxiety, and insomnia among adolescents with FPVEs. Besides, the present study mainly examined the internalizing problems (e.g., depression and anxiety) among adolescents with FPVEs. However, externalizing problems were also of high research significance, which should be further investigated in future research. In addition, the specific age of the onset of victimization may influence the development of psychopathology, given that younger adolescents may possess less affect-regulation and coping strategies. Moreover, only social support and self-compassion were discussed as protective factors in this study, while the roles of other protective factors (e.g., resilience) were not taken into account. Last but not least, the mechanism of these protective factors calls for more in-depth researches, providing more evidence for effective interventions. These are important questions for future studies.

Despite these limitations, utilizing data from a large sample, the study reported the prevalence of FPVEs among Chinese adolescents and mental health problems in this particular cohort. The study also examined some correlated statistically significant risk and protective factors based on the dynamic stress-vulnerability model. This study identified specific implications for the mental health of adolescents with FPVEs. First, the prevalence of depression, anxiety, and insomnia among adolescents with FPVEs in China is relatively high, suggesting that urgent preventive or intervention measures are needed. For example, programs that included interpersonal efficacy training [68] and mindful self-compassion programs [69] may be beneficial to reduce the risk of FPVEs or later negative consequences and improve the overall well-being of the unique group. More attention must also be paid to older adolescents, girls, left-behind children, and adolescents with ACEs. Strengthening social support and SC among adolescents, in general, may alleviate their mental health problems and improve their sleep quality.

## Figures and Tables

**Table 1:** Demographic characteristics of the study participants (N = 490).

Variables	N	%	Variables	N	%
Gender			Father’s education		
Female	234	47.8	Junior high school or below	376	76.7
Male	256	52.2	High school	72	14.7
Age			Undergraduate	28	5.7
11	52	10.6	Above undergraduate	14	2.9
12	74	15.2	Mother’s education		
13	128	26.1	Junior high school or below	360	73.5
14	125	25.5	High school	76	15.5
15	81	16.5	Undergraduate	37	7.6
16	30	6.1	Above undergraduate	18	3.5
Siblings			Monthly income per capita (RMB)		
Single	91	18.6	<1000	77	15.7
Non-single	399	81.4	1000–1999	88	18.0
Residence			2000–2999	101	20.6
Urban	105	21.4	3000–3999	95	19.4
Rural	385	78.6	4000–4999	39	8.0
Migrant			5000–5999	15	3.1
Migrant	37	7.6	≥6000	75	15.3
Non-migrant	453	92.4	Concurrence with other ACEs		
Family structure			1	66	13.5
Intact	388	79.2	2	64	13.1
Other	102	20.8	3	78	15.9
Left-behind Status			4	84	17.1
Left-behind	208	42.4	5 or more	172	35.1
Non-left-behind	282	57.6			

**Table 2:** Multiple linear regression of various independent variables on depression, anxiety, and insomnia symptoms.

	Depression Symptoms			Anxiety Symptoms			Insomnia Symptoms		
**Independent Variables**	**β**	**t**	**∆R^2^**	**β**	**t**	**∆R^2^**	**β**	**t**	**∆R^2^**
**Step 1**									
Demographic Factors			0.07 ^***^			0.05 ***			0.10 ***
Age	0.19	4.32 ***		0.14	3.09 **		0.22	5.10 ***	
Gender	0.20	4.46 ***		0.17	3.86 ***		0.23	5.32 ***	
**Step 2**									
Social Factors			0.13 ***			0.11 ***			0.13 ***
Father’s Education	−0.03	−0.57		−0.07	−1.28		−0.07	−1.37	
Mother Education	0.09	1.80		0.15	2.82 **		0.05	0.88	
Family Structure	0.00	0.04		0.02	0.54		−0.01	−0.15	
Siblings	−0.04	−0.83		−0.02	−0.49		0.01	0.31	
Family Income	−0.04	−1.06		−0.04	−0.85		−0.04	−1.05	
Left-behind Status	−0.12	−2.75 **		−0.12	−2.86 **		−0.08	−2.00 *	
Social Support	−0.33	−7.73 ***		−0.28	−6.46 ***		−0.35	−8.45 ***	
**Step 3**									
Psychological Factors			0.13 ***			0.13 ***			0.06 ***
ACEs	0.13	3.10 **		0.16	3.93 ***		0.09	2.15 *	
PYD	0.04	0.80		0.08	1.82		0.03	0.72	
SC	−0.38	−8.75 ***		−0.37	−8.26 ***		−0.26	−5.93 ***	

Note: * *p* < 0.05, ** *p* < 0.01, *** *p* < 0.001. ACEs: adverse childhood experiences; PYD: positive youth development; SC: self-compassion.

## Data Availability

The data presented in this study are available on request from the corresponding author. The data are not publicly available due to being part of an ongoing project and privacy reasons.
